# Structure‐function analysis of two closely related cutinases from *Thermobifida cellulosilytica*

**DOI:** 10.1002/bit.27984

**Published:** 2021-11-17

**Authors:** Jenny Arnling Bååth, Vera Novy, Leonor V. Carneiro, Georg M. Guebitz, Lisbeth Olsson, Peter Westh, Doris Ribitsch

**Affiliations:** ^1^ Department of Biotechnology and Biomedicine Technical University of Denmark Lyngby Denmark; ^2^ Dept. of Biology and Biological Engineering, Division of Industrial Biotechnology, Wallenberg Wood Science Center Chalmers University of Technology Gothenburg Sweden; ^3^ Institute of Environmental Biotechnology University of Natural Resources and Life Sciences (BOKU) Tulln Austria

**Keywords:** cutinase, enzyme kinetics, PET hydrolase, structure‐function analysis, substrate specificity

## Abstract

Cutinases can play a significant role in a biotechnology‐based circular economy. However, relatively little is known about the structure–function relationship of these enzymes, knowledge that is vital to advance optimized, engineered enzyme candidates. Here, two almost identical cutinases from *Thermobifida cellulosilytica* DSM44535 (Thc_Cut1 and Thc_Cut2) with only 18 amino acids difference were used for a rigorous biochemical characterization of their ability to hydrolyze poly(ethylene terephthalate) (PET), PET‐model substrates, and cutin‐model substrates. Kinetic parameters were compared with detailed in silico docking studies of enzyme‐ligand interactions. The two enzymes interacted with, and hydrolyzed PET differently, with Thc_Cut1 generating smaller PET‐degradation products. Thc_Cut1 also showed higher catalytic efficiency on long‐chain aliphatic substrates, an effect likely caused by small changes in the binding architecture. Thc_Cut2, in contrast, showed improved binding and catalytic efficiency when approaching the glass transition temperature of PET, an effect likely caused by longer amino acid residues in one area at the enzyme's surface. Finally, the position of the single residue Q93 close to the active site, rotated out in Thc_Cut2, influenced the ligand position of a trimeric PET‐model substrate. In conclusion, we illustrate that even minor sequence differences in cutinases can affect their substrate binding, substrate specificity, and catalytic efficiency drastically.

## Graphical abstract

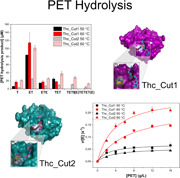

Two structurally almost identical cutinases from *Thermobifida cellulosilytica*, active on the polyester PET, were assayed on PET, PET fragments, and cutin‐model substrates. The enzymes diverge by only 18 amino acids, but these minor sequence differences affected their substrate specificity and catalytic efficiency drastically. To get detailed insights on the structure‐function relationship and to suggest engineering targets for improved performance of these enzymes, the authors compared kinetic parameters and binding isotherms with in‐silico docking studies of enzyme‐ligand interactions.

## 1 INTRODUCTION

Cutinases (EC 3.1.1.74) are relatively small serine esterases (20 to 30 kDa) that belong to the α/β hydrolase superfamily. They possess a Ser–His–Asp catalytic triad and an oxyanion hole for transition state stabilization, and can catalyze hydrolysis, esterification, and transesterification of hydrophobic compounds (Bauer et al., 2020; Chen et al., 2020). In contrast to lipases, the catalytic triad in cutinases is located in a shallow binding cleft, exposed to the solvent, and no surface activation is required. Due to this architecture, cutinases can hydrolyze polymer structures with a high molecular weight (Carr et al., 2020; Chen et al., 2020). Cutinases are secreted by plant pathogens to attack and degrade the hydrophobic apoplastic barrier of higher plants (Chen et al., 2013, 2020), constituted of the polyesters cutin and suberin (Nawrath, 2002). These linear or branched polymers are composed of long chain hydroxy and epoxy fatty acids with glycerol or ferulic acid head groups, and are usually n‐C16 to n‐C18 (cutin) or n‐C16 to n‐C34 (suberin) in length (Nawrath, 2002). Apart from providing a resistance barrier against pathogen infection, cutin and suberin are contributing to the management of water, solute, and gas translocation and increase the physical strength of plant cell walls (Chen et al., 2013; Nyyssölä, 2015). Their multitude of functionalities (including epoxy, alcohol, and carboxy groups, and unsaturated bonds (Nawrath, 2002)) have made cutin‐ and suberin‐derived fatty acids of interest as polymer precursors, replacing conventional fossil oil‐based compounds (Ferrario et al., 2016; Gandini et al., 2006). Due to their hydrolysis, esterification‐ and transesterification activity (Chen et al., 2013), cutinases could be key to facilitating these fine chemical, plant cuticle‐based applications.

The main driver for the recent interest in cutinases, however, is their ability to hydrolyze and modify man‐made polyesters, such as poly(ethylene terephthalate) (PET) and poly lactic acid (PLA) (Chen et al., 2020; Nikolaivits et al., 2018; Pellis et al., 2016; Tournier et al., 2020; Urbanek et al., 2020; Wackett & Robinson, 2020). Exploitation of this at large scale could make cutinases a major player in mitigating the environmental risk connected to plastic accumulation and enable stepwise recovery of valuable polymer building blocks from plastic waste. However, despite recent research (Chen et al., 2020; Nikolaivits et al., 2018; Nyyssölä, 2015; Tournier et al., 2020), the enzyme structure and its impact on activity and substrate specificity is not yet fully understood. The biodiversity is vast, with the Carbohydrate Esterase 5 category alone (in the Carbohydrate‐Active Enzymes Database) containing above 3000 cutinases, with everything between ~10 and ~90% amino acid sequence identity (http://www.cazy.org/; Lombard et al., 2014)).

**Figure 1:**
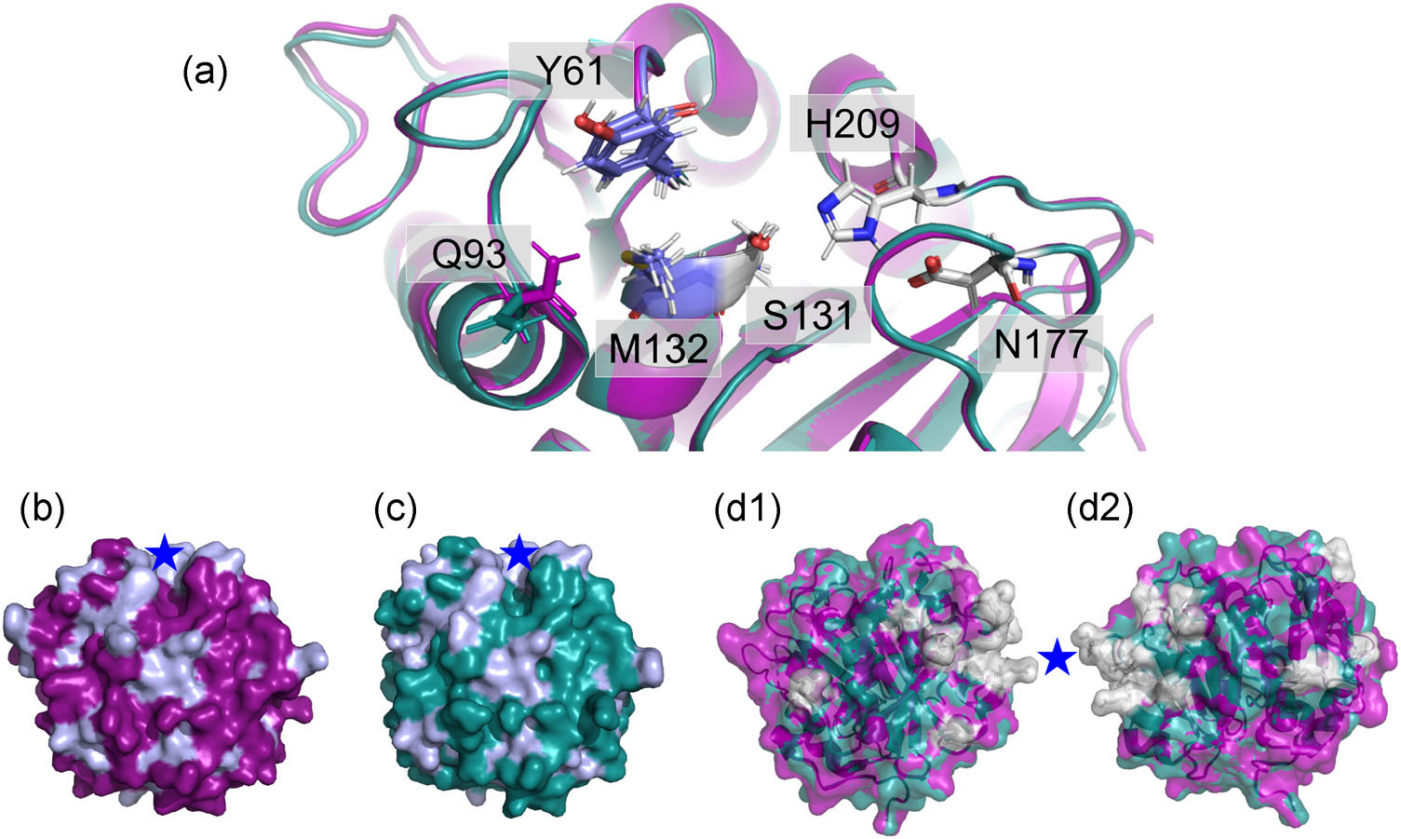
Active site overlay (a), surface models (b, c), and amino acid differences (d1 and d2) of the cutinases Thc_Cut1 (magenta) and Thc_Cut2 (teal). The active site overlay displays the catalytic triad (grey) and the oxyanion hole (blue), as well as the amino acid Q93, which configuration change possibly affect enzyme‐ligand interaction. The surface models show Thc_Cut1 (b) and Thc_Cut2 (c), with hydrophobic residues (Tyr, Phe, Trp, Ala, Val, Ile, Leu, Met) marked in light blue and the binding cleft indicated by a star. Panels d1 and d2 represent overlays of Thc_Cut1 (magenta) and Thc_Cut2 (teal), with amino acid differences marked in grey. Note, d1 and d2 show different angles of the enzyme overlays, representing the “front” and “back” of the enzymes, respectively. “Region 1,” where most of the amino acid differences occur (Table S1) is marked by a star. The amino acid sequence alignment of Thc_Cut1 and Thc_Cut2 can be found in Figure S1

Some microorganisms harbor two or more cutinases, which indicates potential differences in substrate specificity and physiological function. This is the case for *Thermobifida cellulosilytica* DSM44535 that produces two almost identical cutinases, Thc_Cut1 and Thc_Cut2, differing in sequence by only 18 amino acids mainly positioned at the enzymes' surface (Figures 1 and S1; Table S1; Herrero Acero et al., 2013). Thc_Cut1 (PDB: 5LUI) and Thc_Cut2 (PDB: 5LUJ) share identical configurations of the catalytic triad (Ser131; Asp177; His209) and the oxyanion hole (Tyr61; Met132; Figure 1a), with the residue Gln93 close to the active site representing the only configurational difference (Figure 1a). The overall globular structure of the enzymes, the architecture of the binding cleft, as well as the hydrophobic areas are also highly similar (Figure 1b,c). Out of the 18 differing amino acids, 13 are located in one region (Figure 1, panels d1/2; “Region 1” in Table S1). In eight cases (of which five are found in “Region 1”), Thc_Cut2 has replaced a shorter amino acid side chain by a longer one or an aromatic ring (Table S1) that would be oriented parallel to a planar substrate (Figure 1, panels d1/2). Despite sequential and structural similarity, the two enzymes have shown significant differences in activity and substrate preferences (Herrero Acero et al., 2011, 2013; Ribitsch et al., 2017). Thc_Cut1 has been described to be superior in hydrolyzing PET and the aromatic trimer bis(2‐(benzoyloxy)ethyl) terephthalate (BETEB) (chemical structure displayed in Figure S2). Thc_Cut2, on the other hand, performed slightly better on PLA (Herrero Acero et al., 2011; Ribitsch et al., 2017).

Since the two enzymes are highly similar, and are only varying in single, defined amino acid regions, they provide an ideal case for analysis of the structure–function relationship. However, proper kinetic information is lacking, which is required for a deeper understanding. Hence, the aim of this study was to conduct a rigorous biochemical comparison between Thc_Cut1 and Thc_Cut2 to obtain kinetic parameters for a variety of ester substrates of different size and solubility. In a second step, detailed structural analysis and ligand‐docking was performed to connect observed catalytic differences to structural changes between the two enzymes. Substrates used in this study included PET, insoluble and soluble PET‐model substrates, and soluble substrates modelling suberin‐ and cutin‐derived compounds. Herein we identify which structural dissimilarities between Thc_Cut1 and Thc_Cut2 that are most likely responsible for the measured differences in enzyme binding, substrate specificity, and catalytic efficiency. The generated knowledge gives an increased understanding of the structure–function relationship of cutinases and provides a foundation for future rational design of these enzymes, optimizing them for various applications.

## 2 MATERIALS AND METHODS

### 2.1 Substrates and chemicals

Semi‐crystalline PET powder (Product No.: ES306031) was purchased from Goodfellow Co. The crystallinity was reported to be >40% by the supplier and particle size distribution (determined by laser diffraction) ranged from 10 to 300 µm with a mean value of 100 µm. The PET‐model substrate BETEB was synthesized from bis(2‐hydroxyethyl) terephthalate (ETE) and benzoyl chloride as described elsewhere (Arnling Bååth et al., 2021). The model substrate ETE and para‐nitrophenol compounds esterified with fatty acids with increasing chain length (C2 to C16, denoted pNP‐C2, pNP‐C4, pNP‐C8, pNP‐C12, and pNP‐C16) were purchased from Sigma‐Aldrich. PET‐model compounds used as standard samples were purchased from Sigma‐Aldrich (T, B and ETE), enzymatically produced (ET, as described previously (Arnling Bååth et al., 2020)) or kind gifts from Novozymes A/S (BE, BET, BETE, TET, TETE, TETET, ETETETE). Chemical structures and full names are found in Figure S2.

### 2.2 Enzymes

Construction of Thc_cut1 and Thc_cut2 genes, sequencing, protein expression and purification by Ni‐affinity chromatography were performed using methods described previously (Herrero Acero et al., 2013; Ribitsch et al., 2017). Molar enzyme concentrations were determined by Abs280 and the calculated extinction coefficients (Gasteiger et al., 2005). The thermal transition mid‐point (T_m_) was determined by differential scanning fluorimetry using a Nanotemper Prometheus Nt.48 (Nanotemper). Enzyme samples (in phosphate buffer) were heated from 20°C to 99°C at 10% laser intensity and a rate of 1.5°C/min. Resulting melt curves are found in Figure S3. Activities at different pH values were determined for both enzymes in the pH range of 6–8.7. For this, volumetric activities (1 µmol of pNP released per minute and ml) at each pH was measured using 3.125 mM pNP‐C8 as substrate and 50 mM sodium phosphate (pH 6–8) and 50 mM Tris‐HCl (pH 8 and 8.7) as buffers. pH profiles are depicted in Figure S4.

### 2.3 Activity assay with PET and PET‐model substrates

PET and BETEB are insoluble in water and were prepared as suspensions in 50 mM sodium phosphate buffer, pH 8. For determination of activity on PET, a plate reader‐based assay (Abs240) adapted for initial rate kinetics was used (Arnling Bååth et al., 2020, 2021). Reactions (250 µl total volume, in triplicates) were performed in low binding 96‐well plates (Greiner Bio‐One™ 655900), sealed and incubated at 50°C or 60°C, shaking at 450 rpm in an incubator (KS 4000 ic control; IKA). For Michaelis–Menten (MM) analyzes, reactions were performed with 0.06 µM enzyme and 0–16 g·L^−1^ PET with contact times of 1 h (60°C) or 3 h (50°C) to obtain good Abs240 signals but still within the linear range of the progress curve, that is, steady‐state (Figure S5). All reactions were stopped by centrifugation (3 min, 2000*g*) and 100 µl supernatant was transferred to UV‐transparent microplates (Corning) and measured in a plate reader at 240 nm. The pooled product formation was quantified against standards of ETE as described previously (Arnling Bååth et al., 2020). Data were fitted to the nonlinear MM equation using ORIGIN PRO 2019. Reactions with 20 g·L^−1^ PET and 0.1 µM enzyme, incubated over 3 h at either 50°C or 60°C were performed separately for analysis with reversed‐phase high‐performance liquid chromatography (RP‐HPLC), to detect the separate reaction products from enzymatic hydrolysis. These samples were quenched by centrifugation and the addition of concentrated HCl.

The activity assay with BETEB was performed similar to the PET assay, but reaction products were detected with RP‐HPLC due to high background absorbance of BETEB using the plate reader. BETEB reactions were performed in duplicates at 50°C or 60°C for 20 or 15 min, respectively, in an Eppendorf thermomixer operated at 1100 rpm. For MM analysis, the substrate load varied between 0 and 0.5 g·L^−1^ and enzyme concentration was kept low at 0.01 µM (Thc_Cut1) or 0.005 µM (Thc_Cut2) to avoid secondary reaction products. Reactions with 0.5 g·L^−1^ PET and 0.01 µM enzyme, incubated over 20 min at either 50 or 60°C were performed separately to compare product profiles between the enzymes. All samples were quenched by centrifugation followed by addition of concentrated HCl. Kinetic parameters were calculated from quantification of produced BETE, BE, BET, and ET and data were fitted to the nonlinear MM equation using ORIGIN PRO 2019.

ETE is, compared to PET and BETEB, soluble in buffer. However, reactions were similarly performed in low binding 96‐well plates, sealed and incubated at 50°C in an Eppendorf thermomixer operated at 1100 rpm. For MM analysis, the incubation time was 15 min, the substrate load varied between 0 and 2 mM and the enzyme concentration was 0.1 µM. The samples were quenched with concentrated HCl before analysis with RP‐HPLC. Kinetic parameters were calculated from quantification of produced ET and data were fitted to the nonlinear MM equation using ORIGIN PRO 2019.

### 2.4 Binding isotherms

The adsorption of the enzymes to PET was determined using a fixed PET concentration of 50 g·L^−1^ and enzyme concentrations ranging from 0 to 1.5 µM. PET reactions were incubated in low binding 96‐well plates for 1 h and 1000 rpm at either 50 or 60°C. The separation of the solid and liquid phase was done by centrifugation in a preheated centrifuge (set to the incubation temperature). The protein content of the supernatant after incubation with PET was determined using a micro BCA protein kit from Thermo Fischer Scientific (Product No.: 23225), where 100 µl supernatant was mixed with 100 µl freshly prepared BCA working solution. The subsequent color reaction took place in a microtiter plate incubated at 37°C for 2 h and 300 rpm. After incubation with the BCA reagent, 150 µl was transferred to a new microtiter plate and the absorbance was read at 562 nm in a plate reader. Standard curves of the individual enzymes (ranging from 0 to 1.5 µM in concentration) were used to quantify the amount of free enzyme in the reactions. From the amount of free enzymes (*E*_free_) and the total enzyme concentration (*E*_0_), the amount of bound enzyme could be calculated as *E*_bound_ = *E*_0_ – *E*_free_. Data were fitted to a Langmuir isotherm and adsorption parameters extracted using ORIGIN PRO 2019. All experiments were done in triplicates.

### 2.5 Detection of reaction products by RP‐HPLC

The quantification of reaction products from enzymatic hydrolysis of BETEB, ETE and selected PET reactions was determined by RP‐HPLC (Chemstation Series 1100; Hewlett Packard). The instrument was equipped with a diode array detector and an ODS‐L optimal column from Capital HPLC (25 × 4.6 mm) packed with C18 particles 5 µm in diameter size. Injection volume was 20 µl and samples were eluted with 24% acetonitrile over 25 min (for ETE reactions) and over 40 min for BETEB and PET reactions. Products were identified based on absorption at 240 nm. Flow rate was set to 0.5 ml·min^−1^ and the column was kept at 40°C. Peak analysis was performed using the ChemStation for LC 3D software. Standards with known concentrations of T, ET, ETE, B, BE, BET, BETE, TET, TETE, TETET, and ETETETE (Figure S2) were used to quantify reaction products. Duplicates and substrate blanks (for quantification of autohydrolysis) were included for all reactions.

### 2.6 Activity assay with pNP‐substrates

Substrate solutions of pNP‐C2, pNP‐C4, pNP‐C8, pNP‐C12, and pNP‐C16 were prepared in a concentration range of 0.5–250 mM in absolute ethanol, and stored at −20°C. All reactions were performed in 96‐well plates (Greiner Bio‐One™ 675801) with 200 µl total volume. For measuring kinetic properties of Thc_Cut1 and Thc_Cut2 using pNP‐C2, pNP‐C4, pNP‐C8, and pNP‐C12 as substrates, 140 µl of 50 mM sodium phosphate buffer (pH 7, Na‐Ph) was mixed with 50 µl of enzyme solution and preheated to 40°C. The reaction was started by adding 10 µl of substrate solution. Due to reduced solubility, pNP‐C16 solutions and Na‐Ph were preheated to 70°C and then mixed at ratio 1:15 before cooling it down to 40°C. A total of 150 µl thereof was then mixed with 50 µl of enzyme to start the reaction. The release of pNP over time was followed at 405 nm using a SpectroStar Nano (BMG LABTECH) plate reader, and absorbance was quantified against pNP standards. pNP released by autohydrolysis was subtracted by running one control per reaction, replacing the enzyme with Na‐Ph. Activities were derived from the linear area of the progress curve. For calculation of kinetic parameters, the specific activities (1 µmol of pNP released per minute and mg enzyme) were plotted against the respective substrate concentration and fitted with the MM equation using GraphPad Prism.

### 2.7 Docking simulations

Docking simulations were performed using the flexible Glide Standard Precision (SP) function of the Maestro 12.3 program (Schrödinger, Inc.). Thc_Cut1 (PDB: 5LUI), Thc_Cut2 (PDB: 5LUJ) and the substrate molecules, prepared using the ChemDraw software (PerkinElmer), were loaded into the program and adjusted to the experimental pH of 7 using the EPIK module. A receptor grid box with 20 Å axes length, centered at the catalytic serine (Ser131), was generated. It contained information for two docking constraints: a spatial constraint (nuclear Overhauser effect; NOE) to bring the ester bond into at least 3 Å proximity to the catalytic serine; and a hydrogen bond constraint (hbond) to orient the carbonyl oxygen of the ester bond towards the oxyanion hole. For the ligand docking, hydrogen bonds were rewarded, and an EPIK state penalty added to the docking score. Each enzyme was docked with each substrate under 4 constraints: using no constraints, NOE, hbond, or both. Poses were evaluated by the user according to the proximity of the ester oxygen bridge to the catalytic serine, the orientation of the carbonyl oxygen to the oxyanion hole, as well as the Glide Emodel score. For the best pose, the binding energy was then calculated using the Prime MM‐GBSA function in Maestro (Genheden & Ryde, 2015). Overlays and enzyme structures presented herein were prepared using PyMOL 4.6 (Schrödinger).

## 3 RESULTS

### 3.1 The degradation mechanism of PET and PET‐model substrates

To analyze differences in hydrolysis of Thc_Cut1 and Thc_Cut2 towards the aromatic polymer PET and smaller aromatic PET oligomers, kinetic analyzes on semi‐crystalline PET and the two model substrates BETEB (aromatic trimer, where B, E, and T represent benzoic acid, ethylene glycol, and terephthalic acid, respectively) and ETE (bis(2‐hydroxyethyl) terephthalate; aromatic monomer) were performed (chemical structures are found in Figure S2). BETEB is water insoluble like PET, whereas ETE is soluble and a potential reaction product from enzymatic PET and BETEB hydrolysis. In addition to steady‐state kinetics, the adsorption of the two enzymes to the PET substrate was determined by traditional binding isotherms and the difference in released products from PET hydrolysis was determined by RP‐HPLC.

#### 3.1.1 Steady‐state kinetics and binding parameters on semicrystalline PET

**Figure 2:**
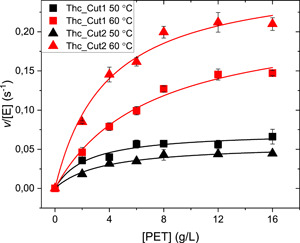
MM plots for Thc_Cut1 (squares) and Thc_Cut2 (triangles) with initial hydrolysis rate as a function of PET load. Symbols are experimental data from reactions at 50°C (black) or 60°C (red) with 0.1 µM enzyme load. Error bars represent standard deviations of triplicate measurements. Lines represent the best fit of the nonlinear MM equation. MM, Michaelis‐Menten; PET, poly(ethylene terephthalate)

Kinetic parameters and corresponding MM plots from enzymatic hydrolysis of PET at 50°C and 60°C are presented in Table 1 and Figure 2. At 50°C, Thc_Cut1 showed a more efficient PET hydrolysis as compared to Thc_Cut2, with a 1.5‐fold lower *K*_M_ and a 1.3‐fold higher substrate turnover (*k*_cat_) (Table 1). However, Thc_Cut2 benefits more from increased temperature and becomes superior at 60°C, where it showed a 2.3‐fold higher *k*_cat_/*K*_M_ as compared to Thc_Cut1. The improvement of Thc_Cut2 over Thc_Cut1 at 60°C was mainly caused by a 1.8‐fold lower *K*_M_ (Table 1). This temperature‐dependent behavior is not reflected by their respective melting temperatures (*T*_m_) (Figure S3) and hence probably not a result of different thermostability. The enhanced substrate turnover with temperature is not paralleled by an improved (lower) *K*_M_ for any of the enzymes, and for Thc_Cut1, *K*_M_ is even increasing. The pronounced improvement of k_cat_ at higher temperature, especially for Thc_Cut2, is possibly a result of the increased mobility of the polymer chains when approaching the glass transition temperature (*T*_g_) of PET, which is approximately 70°C (Shirke et al., 2016).

**Table 1:** Kinetic parameters of Thc_Cut1 and Thc_Cut2 on semi‐crystalline PET^a^

T (°C)	^mass^K_M_ (g·L^−1^)	k_cat_ (s^−1^)	^mass^k_cat_/K_M_ (L·g^−1^·s^−1^)
		Thc_Cut1	
50	2.3 ± 0.68	0.072 ± 0.0056	0.031 ± 0.0094
60	6.9 ± 1.2	0.22 ± 0.017	0.032 ± 0.0061
		Thc_Cut2	
50	3.5 ± 0.68	0.057 ± 0.0036	0.016 ± 0.0033
60	3.8 ± 0.72	0.27 ± 0.017	0.072 ± 0.014

^a^ Data represent average values and the spread of triplicate experiment.

**Figure 3:**
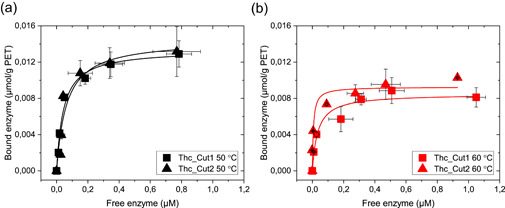
Adsorption isotherms for Thc_Cut1 (squares) and Thc_Cut2 (triangles) on semicrystalline PET, with a fixed load of 50 g·L^−1^ and a contact time of one hour at (a) 50°C (black) and (b) 60°C (red). Enzyme loads range from 0 to 1.5 µM. Error bars represent SDs of triplicate measurements. Lines represent the best fit of the nonlinear Langmuir equation. At 50°C, the affinity and the maximum binding capacity of the two enzymes is similar, whereas Thc_Cut2 display a stronger affinity for PET at 60°C compared to ThC_Cut1, seen from the steeper initial curve in (b). PET, poly(ethylene terephthalate)

Binding parameters of Thc_Cut1 and Thc_Cut2 to PET are presented in Table 2, extracted from the adsorption isotherms in Figure 3. As observed from these data, the enzymes share similar maximal binding capacity (Γ_max_) to the PET surface at both temperatures. There is no clear difference in affinity (*K*_d_) between the two enzymes at 50°C. However, Thc_Cut2 displayed a significantly greater affinity to PET than Thc_Cut1 at 60°C, with a 4.1‐fold lower *K*_d_. This agrees with corresponding *K*_M_ values at this temperature for the respective enzyme.

**Table 2:** Binding parameters on semi‐crystalline PET at 50 or 60°C derived from experiments shown in Figure 3, a

	K_d_ (µM)	Γ_max_(µmol·g−1)
Thc_Cut1 50°C	0.044 ± 0.0074	0.014 ± 5.7E‐4
Thc_Cut1 60°C	0.012 ± 6.1E‐7	0.0085 ± 5.4E‐4
Thc_Cut2 50°C	0.063 ± 0.024	0.014 ± 0.0016
Thc_Cut2 60°C	0.0029 ± 8.2E‐7	0.0093 ± 5.0E‐4

^a^ Data represent average values and the spread of triplicate experiment.

#### 3.1.2 Product release from enzymatic PET hydrolysis

**Figure 4:**
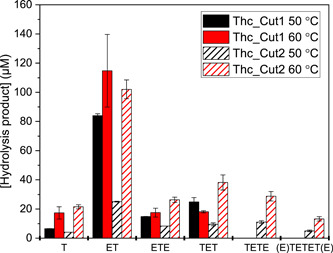
Product quantification from RP‐HPLC analysis of PET hydrolysis by ThC_Cut1 (filled bars) and ThC_Cut2 (striped bars) over 3 h at 50 (black) or 60°C (red) with 20 g·L^−1^ PET and 0.1 µM enzyme. The corresponding RP‐HPLC chromatograms are seen in Figure S6. The products detected were species with one aromatic ring (T, ET, ETE), two aromatic rings (TET/E) and three aromatic rings (E/TETET/E). Chemical structures of these hydrolysis products are seen in Figure S2. Error bars represent SDs of duplicate measurements. PET, poly(ethylene terephthalate); RP‐HPLC, reversed‐phase high‐performance liquid chromatography

To further explain differences in PET hydrolysis between the two enzymes, their respective PET hydrolysis product profiles were investigated by RP‐HPLC (Figures 4 and S6). In agreement with kinetic data, Thc_Cut2 outperformed Thc_Cut1 at the higher reaction temperature of 60°C in terms of total product release, whereas Thc_Cut1 was better at 50°C (Figure 4). The product profiles additionally showed that the enzymes do not only differ regarding rates but also in terms of product diversity. Both enzymes generated the monoaromatic compound ET as main product. However, when comparing the generation of ET and other, larger PET oligomers (species with two and three aromatic rings), Thc_Cut1 showed a higher ET to oligomer ratio, whereas Thc_Cut2 generated more of the larger reaction products. This suggests that Thc_Cut1 prefers hydrolysis of primary reaction products with one to a few aromatic rings in the bulk phase, and interpretation was supported by the superiority of Thc_Cut1 in the hydrolysis of the small and soluble substrate ETE (see below).

#### 3.1.3 Steady‐state kinetics on PET‐model substrates

BETEB (aromatic trimer, Figure S2) is a commonly used PET‐model substrate for characterization of PET hydrolases that have been included in earlier studies of Thc_Cut1 and Thc_Cut2 (Herrero Acero et al., 2011, 2013). However, detailed kinetic data have hitherto not been reported and we therefore assayed the two enzymes on BETEB using the same methodology as for PET. Kinetic parameters extracted from these experiments are found in Table 3, with corresponding MM plots being displayed in Figure S7. Here, Thc_Cut2 had a significantly (1.5‐ to 1.9‐fold) higher turnover rate than Thc_Cut1 at both temperatures, which is in contrast to PET hydrolyzes, where Thc_Cut2 only was superior at 60°C. The product profiles from enzymatic BETEB hydrolysis were similar between the enzymes in terms of product ratios, with BET and BE as main products (Figure S8). This suggests a more *endo*‐acting mode of these enzymes, i.e. a preference for the inner ester bond of the substrate. The turnover rates for both enzymes on BETEB (Table 3) were remarkably higher (100‐fold) as compared to when acting on PET (Table 1). Additionally, the thermoactivation was more moderate on BETEB compared to PET reactions, supporting the notion that pronounced increase in turnover of Thc_Cut2 on PET is an effect of changes in polymer properties close to *T*_g_.

**Table 3:** Kinetic parameters of Thc_Cut1 and Thc_Cut2 on BETEB^a^

	^mass^K_M_ (g·L^−1^)	k_cat_ (s^−1^)	^mass^k_cat_/K_M_ (L·g^−1^·s^−1^)
Thc_Cut1 50°C	0.040 ± 0.0071	4.0 ± 0.18	100 ± 18
Thc_Cut1 60°C	0.049 ± 0.0064	6.2 ± 0.20	130 ± 17
Thc_Cut2 50°C	0.082 ± 0.011	7.4 ± 0.34	90 ± 13
Thc_Cut2 60°C	0.062 ± 0.0056	9.4 ± 0.26	150 ± 14

^a^ Kinetic parameters were calculated from quantification of produced BETE, BE, BET, and ET. Data represent average values and the spread of duplicate experiment.

ETE is a monoaromatic and water‐soluble compound that is a reaction product from enzymatic PET hydrolysis. Kinetic data from ETE hydrolysis are depicted in Table 4 and Figure S9. Here, Thc_Cut 1 was far superior in terms of catalytic efficiency, whereas reactions with Thc_Cut2 could not be saturated up to 2 mM ETE. As mentioned above, the superiority of Thc_Cut1 in hydrolyzing ETE is mirrored in the product profile from enzymatic PET hydrolysis (Figure 4), where Thc_Cut1 more effectively hydrolyzes soluble, primary reaction products than Thc_Cut2.

**Table 4:** Kinetic parameters of Thc_Cut1 and Thc_Cut2 on ETE, pNP‐C2, pNP‐C4, pNP‐C8, pNP‐C12, and pNP‐C16^a^

	K_M_ (mM)	k_cat_ = ^conv^V_max_/E_0_ (s^−1^)	k_cat_/K_M_ (mM^−1^·s^−1^)
Thc_Cut1			
ETE	0.99 ± 0.20	2.6 ± 0.26	2.7 ± 0.61
pNP‐C2	2.31 ± 0.57	0.34 ± 0.02	0.15 ± 0.03
pNP‐C4	0.25 ± 0.01	0.17 ± 0.00	0.70 ± 0.03
pNP‐C8	0.31 ± 0.02	0.21 ± 0.00	0.66 ± 0.04
pNP‐C12	0.07 ± 0.02	0.08 ± 0.00	1.19 ± 0.30
pNP‐C16	0.03 ± 0.00	0.10 ± 0.02	3.18 ± 0.45
Thc_Cut2			
ETE	ND^b^	ND	0.059 ± 0.0012^c^
pNP‐C2	4.56 ± 0.30	0.44 ± 0.03	0.10 ± 0.01
pNP‐C4	0.43 ± 0.11	0.16 ± 0.01	0.38 ± 0.14
pNP‐C8	0.40 ± 0.03	0.26 ± 0.01	0.63 ± 0.01
pNP‐C12	0.05 ± 0.01	0.10 ± 0.02	1.90 ± 0.50
pNP‐C16	0.07 ± 0.01	0.09 ± 0.01	1.40 ± 0.22

^a^ Data represent average values and the spread of duplicate experiment.

^b^ ND, Not detected; could not be saturated up to 2 mM ETE.

^c^ Calculated from the slope of linear regression.

### 3.2 Steady‐state kinetics on cutin‐ and suberin‐model substrates

In addition to PET and PET‐model substrates, kinetic analyzes of Thc_Cut1 and Thc_Cut2 were performed on pNP‐substrates with consecutively longer fatty acid chains, with results being displayed in Table 4. These substrates resemble ferulic acid esterified with fatty acids of different chain lengths, a compound commonly found in both cutin and suberin (Nawrath, 2002), making them suitable model substrates for cutinases (Chen et al., 2013). For both enzymes, the turnover rate is decreasing with increasing chain lengths (from 0.34 and 0.44 to 0.10 and 0.90 s^−1^ for Thc_Cut1 and Thc_Cut2, respectively). Substrate affinity showed the opposite trend, with a falling K_M_ for both enzymes (2.31 to 0.03 and 4.56 to 0.07 mM for Thc_Cut1 and Thc_Cut2, respectively). Both enzymes had the highest catalytic efficiency for the longest fatty acid chains at 3.18 mM^−1^·s^−1^ (Thc_Cut1) and 1.40 mM^−1^·s^−1^ (Thc_Cut2). As the pNP‐C16 strongly resembles feruloyl palmitate, a compound abundantly present in both cutin and suberin, this result indicates that the enzymes show a preference for the more native‐like substrates. Similar to results acquired using the soluble aromatic substrate ETE, Thc_Cut1 in general has lower K_M_ values and better catalytic efficiency on the smaller pNP‐substrates as compared to Thc_Cut2 (Table 4).

### 3.3 Analysis of enzyme‐substrate interaction by in silico docking experiments

**Figure 5:**
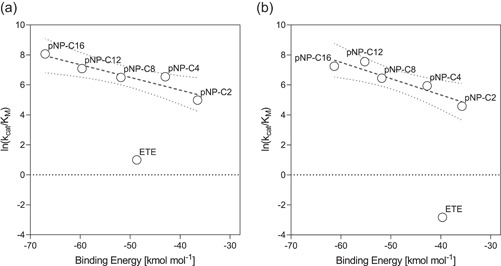
Correlation between catalytic efficiency and binding energies of the soluble substrates ETE and pNP‐C2 to C16 with Thc_Cut1 (a) and Thc_Cut2 (b). Kinetic parameters can be found in Table 4, and binding energies are listed in Table S2. The linear regression is calculated for the pNP‐substrates and is indicated by the dashed line. The dotted lines indicate the 95% confidence band

**Figure 6:**
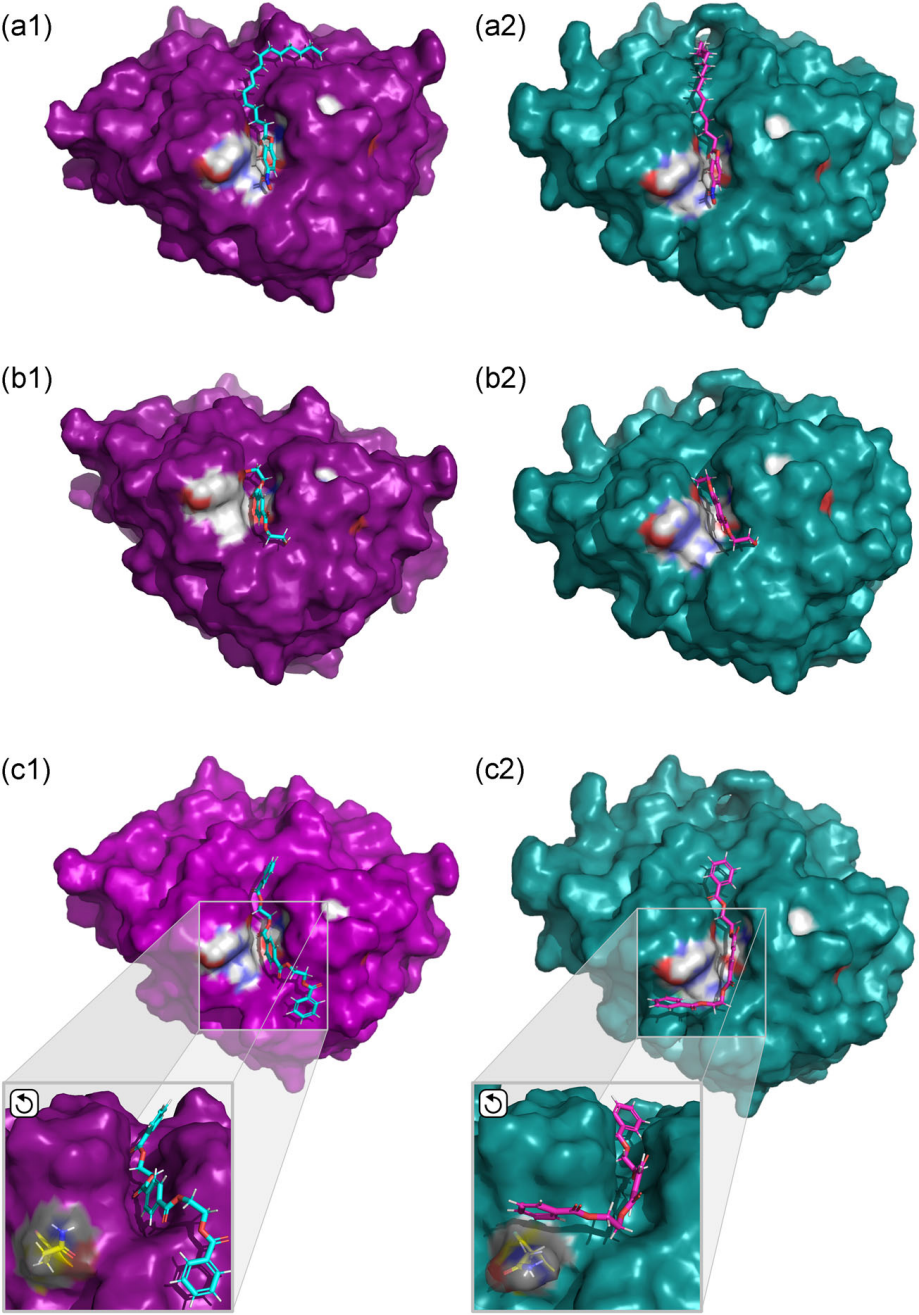
Docked structures of Thc_Cut1 (magenta, a/b/c‐1) and Thc_Cut2 (teal, a/b/c‐2) with pNP‐C16 (a‐1/2) and the PET‐model substrates ETE (b‐1/2) and BETEB (c‐1/2). Depicted are surface models with the catalytic triad marked in grey and the oxyanion hole marked in blue, as introduced in Figure 1. The position and effect of amino acid residue Q93 (yellow) in BETEB docking is visualized in the enlarged portion of panels c‐1/2. The ligands are shown in turquois (Thc_Cut1, a/b/c‐1) and pink (Thc_Cut2, a/b/c‐2). Angles were adjusted for clarity

To obtain insight on enzyme‐ligand interaction, docking analysis was performed using Thc_Cut1 and Thc_Cut2 and all the smaller substrates BETEB, ETE, and pNP‐C2 to C16 that were included in the biochemical characterization. The calculated binding energies and the corresponding docking scores are summarized in Table S2. Figure 5 shows the logarithmic correlation between the catalytic efficiency and the binding energy for the soluble substrates (where parameters in molar units are available). Figure 6 highlights the differences in the docked structures of Thc_Cut1 and Thc_Cut2 with pNP‐C16, ETE and BETEB.

#### 3.3.1 Docking of the soluble substrates ETE and pNP‐fatty acids, and their correlation to the catalytic efficiency

From docking analysis of the soluble substrates, the pNP‐substrates represent one system with the same head group and mode of binding around the ester bond. As seen in Figure 5, for both enzymes, a linear correlation between binding energy and ln(*k*_cat_/*K*_M_) was observed when plotting these parameters for the pNP‐substrates. The slope of the resulting regression is slightly steeper for Thc_Cut2 (−0.11; *R* ^2^ 0.88) than for Thc_Cut1 (−0.09; *R* ^2^ 0.87), which was mainly caused by the better (lower) binding energy of Thc_Cut1 towards pNP‐C12 and C16. Examining the enzyme‐ligand interaction closer (Figure 6; panels a1 and a2), the change in the interaction with the longer chain substrate was likely caused by different configurations in an area adjacent to the active site cleft. In the case of Thc_Cut2, the aliphatic chain continued straight over the enzyme, threading into a tunnel that is only apparent in the surface model of Thc_Cut2 (Figure 6, panel a2). In the case of Thc_Cut1, the chain bends over, folding into a groove‐like structure. This more stable position might have contributed to the higher catalytic efficiency in pNP‐C16 conversion for Thc_Cut1 (Figure 5). The difference in ligand interaction between the two enzymes with all pNP‐substrates can be seen in Figure S10.

When docking ETE, binding energies of −44.3 and −36.5 kcal·mol^−1^ were obtained for Thc_Cut1 and Thc_Cut2, respectively (Table S2). As seen in Figure 5, the results for ETE fall significantly below the linear regression line of the pNP‐substrates. That implies that the catalytic efficiency of the aromatic substrate is much lower than for an aliphatic substrate with comparable binding strength. Figure 5 additionally shows that the 2.9‐fold higher ln(*k*_cat_/*K*_M_) for Thc_Cut1 on ETE as compared to Thc_Cut2 only is matched by a 1.2‐fold increase in binding energy (Table 4 and Figure 5). Closer examination of the docked structure does not show significant pose differences (Figure 6; panels b1 and b2). To further investigate potential variation between the two enzymes and their interaction with ETE, we also docked its reaction product terephthalic acid (“T”; Figure S2). Here, the binding was significantly poorer for Thc_Cut1 (+2.6 kcal·mol^−1^; Table S3) as compared to Thc_Cut2 (−0.7 kcal·mol^−1^), indicating that a faster departure of the product from the active of Thc_Cut1 side may favor the overall reaction rate (Kakaei et al., 2019).

#### 3.3.2 Docking of the insoluble substrate BETEB

We also subjected BETEB to docking analyzes, but due to its insoluble nature and therefore mass‐based kinetic parameters, it is not included in Figure 5. Here, Thc_Cut1 showed an energetically more favorable binding (−58.6 kcal·mol^−1^, Table S2) than Thc_Cut2 (−50.12 kcal·mol^−1^). Although this 1.2‐fold improvement in binding energy was not reflected by a similar increase in ln(*k*_cat_/*K*_M_) (1.02‐ and 1.03‐fold for 50°C and 60°C, respectively, Table 3), a significant drop in K_M_ was observed (Table 3). Looking at the docked structure in more detail, the two enzymes gave two completely different poses (Figure 6; panels c1 and c2). Whereas BETEB aligns straight over the binding cleft in Thc_Cut1, it is bent over perpendicularly in Thc_Cut2, where a large portion of the oligomer is in close contact with residue Q93. As illustrated in Figure 1A, this is due to different positional configuration of the amino acid residue, rotated out in Thc_Cut2. The alignment of Q93 also contributed to different docking results when all potential BETEB hydrolysis products were docked, as described below.

#### 3.3.3 Modelling of the BETEB degradation pathway

Combining the structural insights of Thc_Cut1 and Thc_Cut2 (Figures 1 and 6) with the analysis of BETEB degradation products from Thc_Cut1 and Thc_Cut2 detected by RP‐HPLC (Figure S8), we further tried to understand enzyme‐specific differences and similarities in the BETEB degradation pathway by docking all the degradation products (Table S3 and Figure S11). In the docking experiments, both Thc_Cut1 and Thc_Cut2 exclusively (i.e., under any of the applied constraints) cut the second, inner ester bond in BETEB (Figure S11, Reaction A). Confirming this, BET and BE were the two main reaction products from enzymatic hydrolysis experiments (Figure S8). An efficient conversion of BETEB to BET is further supported for both enzymes by the high binding energies of BETEB (Figure S11 and Table S3). Since none of the enzymes preferred the outer ester bond of BETEB, BETE was not seen as a product in the in silico experiments. Although small amounts of BETE were detected in the actual reactions (Figure S8), the quantities were much lower as compared to BET, again supporting the in silico data. When docking BETE (Figure S11, Reactions E and F), only one acceptable pose was achieved with Thc_Cut1, cleaving the inner ester bond resulting in ET (Reaction F). In agreement with this, ET was detected in small amounts in the experimental set‐up.

Continuing the BETEB degradation pathway from BET, Thc_Cut1 again exclusively cleaved the inner ester bond, resulting in BE and T (Figure S11, Reaction B). Thc_Cut2 also preferred that bond but could also cleave the outer ester bond (Reaction C), resulting in ET and B. BE finally reacts to B and E (Reaction D). In the RP‐HPLC results, B and BE were detected products, but neither T nor ET could be observed. This is likely due to the low enzyme‐substrate ratio and the short reaction time, capturing mainly the first step (Reaction A) of the reaction cascade.

## 4 DISCUSSION

The two wild‐type enzymes from *T. cellulosilytica* DSM44535 investigated in this study, are highly similar in amino acid sequence and have close to identical 3D structures. As a result, Thc_Cut1 and Thc_Cut2 shared several catalytic properties. However, the enzymes also displayed distinct differences in substrate specificity and kinetic behavior, likely due to small amino acids changes at the enzymes' surfaces, as will be discussed hereinafter.

### 4.1 Thc_Cut1 and Thc_Cut2 are efficient PET hydrolases and promising degraders of cutin and suberin

Thc_Cut1 and Thc_Cut2 both possess a surface exposed active site in a shallow cleft, typical for cutinases. This enables them to accommodate and hydrolyze insoluble and bulky ester substrates, which was herein proven by their ability to hydrolyze the aromatic polyester PET and the PET‐model substrate BETEB. The good catalytic properties for the two enzymes on PET and in particular BETEB, make both of them promising PET hydrolases. Further, Thc_Cut1 and Thc_Cut2 appeared to be primarily *endo*‐acting, indicated by a preference towards the inner ester bond of the model substrate BETEB. The *endo*‐activity agrees well with earlier observations for these two enzymes as well as the principal mode of action suggested for other cutinases on BETEB and PET (Eberl et al., 2009; Herrero Acero et al., 2011; Wei et al., 2019).

Apart from being active on PET and PET‐model compounds, Thc_Cut1 and Thc_Cut2 displayed promising activity on aliphatic pNP‐substrates of various length. Such substrate promiscuity of cutinases has been described before (Danso et al., 2019; Nyyssölä, 2015). Here, both enzymes displayed a high catalytic efficiency on the long chain pNP‐substrates. This agrees with the similarity of these model substrates to native fatty acids present in cutin and suberin (Graca & Pereira, 2000; Nawrath, 2002). The outstanding catalytic efficiency on pNP‐C16 render Thc_Cut1 and Thc_Cut2 promising candidates for generating high‐value fatty acids from suberin‐ or cutin‐rich biomasses, such as bark or vegetable residues. Using enzymes for this task would provide a green process, which is in contrast to conventional extraction procedures that rely on severe reaction conditions and the use of harsh chemicals (Ferreira et al., 2012; Graca & Pereira, 2000).

### 4.2 Functional differences between Thc_Cut1 and Thc_Cut2 and their potential physiological role

When analyzing the product distribution of PET degradation by Thc_Cut1 and Thc_Cut2 in detail (Figure 4), dissimilar product profiles of the two enzymes were observed. Hence, Thc_Cut1 is the better enzyme in generating smaller hydrolysis products, possibly due to a superior capacity to hydrolyze small, soluble products released by the primary attack on the PET surface. We could support that hypothesis by kinetic analysis of ETE hydrolysis, both a model compound and a potential hydrolysis product of PET, where Thc_Cut1 performed significantly better than Thc_Cut2. In line with our results, it was reported in a previous study, that the stepwise hydrolysis of BETEB proceeded further when using Thc_Cut1 as compared to Thc_Cut2 (Herrero Acero et al., 2011).

The differences in substrate specificities of Thc_Cut1 and Thc_Cut2 could further suggest a physiological function of these enzymes in the host organism *T. cellulosilytica*. Since Thc_Cut2 seems to produce bigger, insoluble fragments, which are efficiently degraded by Thc_Cut1, the two enzymes might act cooperatively with each other. Identifying and exploiting such cooperative effects between cutinases would further be of great interest for industrial PET recycling.

### 4.3 The effect of differences in surface properties on substrate specificity

The different electrostatic and hydrophobic surface properties of Thc_Cut1 and Thc_Cut2 have previously been described as a reason for the two enzymes' various ability to degrade PET, PLA, and BETEB (Herrero Acero et al., 2011, 2013; Ribitsch et al., 2017). By investigating the enzymes' amino acid sequence and architecture in more detail, we identified that most of the amino acid changes between Thc_Cut1 and Thc_Cut2 are located at the surface and are accumulated in one area (denoted “Region 1”). As highlighted in Figure 1 and Table S1, in “Region 1,” Thc_Cut2 has replaced shorter amino acids found in Thc_Cut1 by larger or aromatic residues. We speculate that these longer amino acid residues could contribute to the “thermoactivation” of Thc_Cut2 on PET substrates (Figures 2 and 3). Although Thc_Cut1 also benefited from the higher temperature, Thc_Cut2 showed a remarkably increased catalytic efficiency and affinity at 60°C, surpassing Thc_Cut1. At a temperature close to T_g_ of PET, the longer amino acids might aid Thc_Cut2 to align and interact with the increasingly mobile aromatic chains of PET, both increasing productive binding and catalytic efficiency drastically. Because PET recycling is performed at temperatures close to *T*_g_ of the polymer, this “thermoactivation” of Thc_Cut2 is highly relevant in an industrial context. When just comparing the two enzymes at 50°C, as was also done previously (Herrero Acero et al., 2011), Thc_Cut1 would emerge as the better PET hydrolase.

### 4.4 The architecture of areas adjacent to the binding cleft influences substrate‐ligand interaction

Variation in the architecture of the enzyme surface adjacent to the catalytic cleft (Figure 1) affected the position of long chain aliphatic ligands and their interaction with the enzyme (Figure 6). A tunnel in Thc_Cut2, instead of an open cleft as observed in Thc_Cut1, might contribute to a reduced fit for the long chain aliphatic substrate, resulting in lower binding energies and catalytic efficiency (Table 4). Considering that most natural occurring cuticle‐derived fatty acids will have a chain length of C16 and longer (Graca & Pereira, 2000; Nawrath, 2002), this change in surface configuration between Thc_Cut1 and Thc_Cut2 might be of interest for developing efficient enzymes for releasing cutin‐ or suberin‐derived fatty acids from various biomasses. Indeed, cutinases shown to efficiently interact with bulky, hydrophobic compounds, for example, the structure‐resolved cutinase from *Cryptococcus* sp. (Kodama et al., 2009) or the suberinase from *Streptomyces scabiei* (Jabloune et al., 2020), have a broad open face around the binding cleft.

### 4.5 The position of residue Q93 is key for interaction with longer aromatic substrates

The active site architecture of the two cutinases looks identical apart from the configuration of residue Q93, which is rotated out in Thc_Cut2 (Figure 1). The importance of this small positional change became obvious in the docking analysis of BETEB, which gave a completely different enzyme‐ligand configuration between the two enzymes (Figure 6). It is possible that this small configurational change is responsible for the better affinity of Thc_Cut1 to BETEB as compared to Thc_Cut2 (Table 3). The role of Q93 seems to be relevant only for longer polymers, as no positional difference was observed for the shorter substrate ETE (Figure 6). In in silico experiments, the configuration of Q93 further enabled Thc_Cut2 to catalyze both the inner and the outer ester bond in the first BETEB degradation product BET (Figure S11, Reactions B and C). Although this remains to be confirmed experimentally, these results might indicate that Q93 plays a role in assigning ester bond preferences in cleavage of long chain aromatic substrates.

## 5 CONCLUSIONS

Due to their sequence and architectural similarity, resolved crystal structures, and proven difference in substrate specificity, the cutinases Thc_Cut1 and Thc_Cut2 represent an ideal case for investigating structure–function relationship for this important enzyme class. By combining extensive biochemical characterization with in silico docking simulations, this study shows that small amino acid changes can be responsible for drastic changes in substrate specificity and catalytic efficiency. Hence, the highlighted area on the Thc_Cut enzymes' surface (“Region 1”) could play an essential role in facilitating efficient interactions with PET at temperatures close to *T*_g_. Similarly, the position of one single amino acid (Q93) adjacent to the binding cleft impacts the ester bond preference in long‐chain polyaromatic substrates. Finally, the area adjacent to the active site can affect the enzyme's interaction with long chain aliphatic substrates, and hence the ability to attack bulky, hydrophobic substrates, such as those presented by the plants' outer skin. Apart from the imminent advantage of providing clear engineering targets, this study also suggests that Thc_Cut1 and Thc_Cut2 could be working cooperatively in degrading PET substrates. Identifying, characterizing, and exploiting such synergism does not only provide the possibility of enhancing time‐space yields in PET recycling applications, but might also provide an explanation to why the organism *T. cellulosilytica* harbors two so closely related enzymes.

## Supporting information

Supporting information.

## Data Availability

All data that support the findings of this study are included in the published article and its Supporting information file.
