# Supplementary material for: Structure‐function analysis of two closely related cutinases from Thermobifida cellulosilytica
Source: Biotechnol Bioeng. 2021 Nov 17;119(2):470–81. doi: 10.1002/bit.27984 (PMC9299132; doi:10.1002/bit.27984)
Supplement: Supplementary file 1 — Supporting information. [file BIT-119-470-s001.pdf]

Supporting information

Structure-function analysis of two closely related cutinases from *Thermobifida cellulosilytica*

Jenny Arnling Bååth[1*], Vera Novy[2*], Leonor Vieira Carneiro[2], Georg M. Guebitz[3], Lisbeth Olsson[2], Peter Westh[1], and Doris Ribitsch[3†]

[1] Department of Biotechnology and Biomedicine, Technical University of Denmark, Søltofts Plads, DK-2800 Kgs. Lyngby, Denmark

[2] Wallenberg Wood Science Center, Division of Industrial Biotechnology, Dept. of Biology and Biological Engineering, Chalmers University of Technology, Kemivägen 10, SE-412 96 Gothenburg, Sweden

[3] Institute of Environmental Biotechnology, University of Natural Resources and Life Sciences (BOKU), Konrad Lorenz Straße 20, 3430 Tulln, Austria

[*]Contributed equally

[†]Corresponding author

Supporting material includes:

Figures S1-S11: Pages S2 to S12

Tables S1-S3: Pages S13 to S15

```
EMBOSS_001         1 MANPYERGPNPTDALLEASSGPFSVSEENVSRLSASGFGGGTIYYPRENN     50
                     ||||||||||||||||||.|||||||||..||..|.||||||||||||||
EMBOSS_002         1 MANPYERGPNPTDALLEARSGPFSVSEERASRFGADGFGGGTIYYPRENN     50

EMBOSS_001        51 TYGAVAISPGYTGTEASIAWLGERIASHGFVVITIDTITTLDQPDSRAEQ    100
                     ||||||||||||||:||:|||||||||||||||||||.||||||||||.|
EMBOSS_002        51 TYGAVAISPGYTGTQASVAWLGERIASHGFVVITIDTNTTLDQPDSRARQ    100

EMBOSS_001       101 LNAALNHMINRASSTVRSRIDSSRLAVMGHSMGGGGTLRLASQRPDLKAA    150
                     |||||::|||.|||.|||||||||||||||||||||||||||||||||||
EMBOSS_002       101 LNAALDYMINDASSAVRSRIDSSRLAVMGHSMGGGGTLRLASQRPDLKAA    150

EMBOSS_001       151 IPLTPWHLNKNWSSVTVPTLIIGADLDTIAPVATHAKPFYNSLPSSISKA    200
                     |||||||||||||||.||||||||||||||||.|||:|||||||:|||||
EMBOSS_002       151 IPLTPWHLNKNWSSVRVPTLIIGADLDTIAPVLTHARPFYNSLPTSISKA    200

EMBOSS_001       201 YLELDGATHFAPNIPNKIIGKYSVAWLKRFVDNDTRYTQFLCPGPRDGLF    250
                     ||||||||||||||||||||||||||||||||||||||||||||||||||
EMBOSS_002       201 YLELDGATHFAPNIPNKIIGKYSVAWLKRFVDNDTRYTQFLCPGPRDGLF    250

EMBOSS_001       251 GEVEEYRSTCPFALE    265
                     |||||||||||||||
EMBOSS_002       251 GEVEEYRSTCPFALE    265
```

Figure S1. Amino acid sequence alignment of Thc_Cut1 and Thc_Cut2. Amino acid differences are marked in yellow, the catalytic triad in blue, and the residues constituting the oxyanion hole in green.

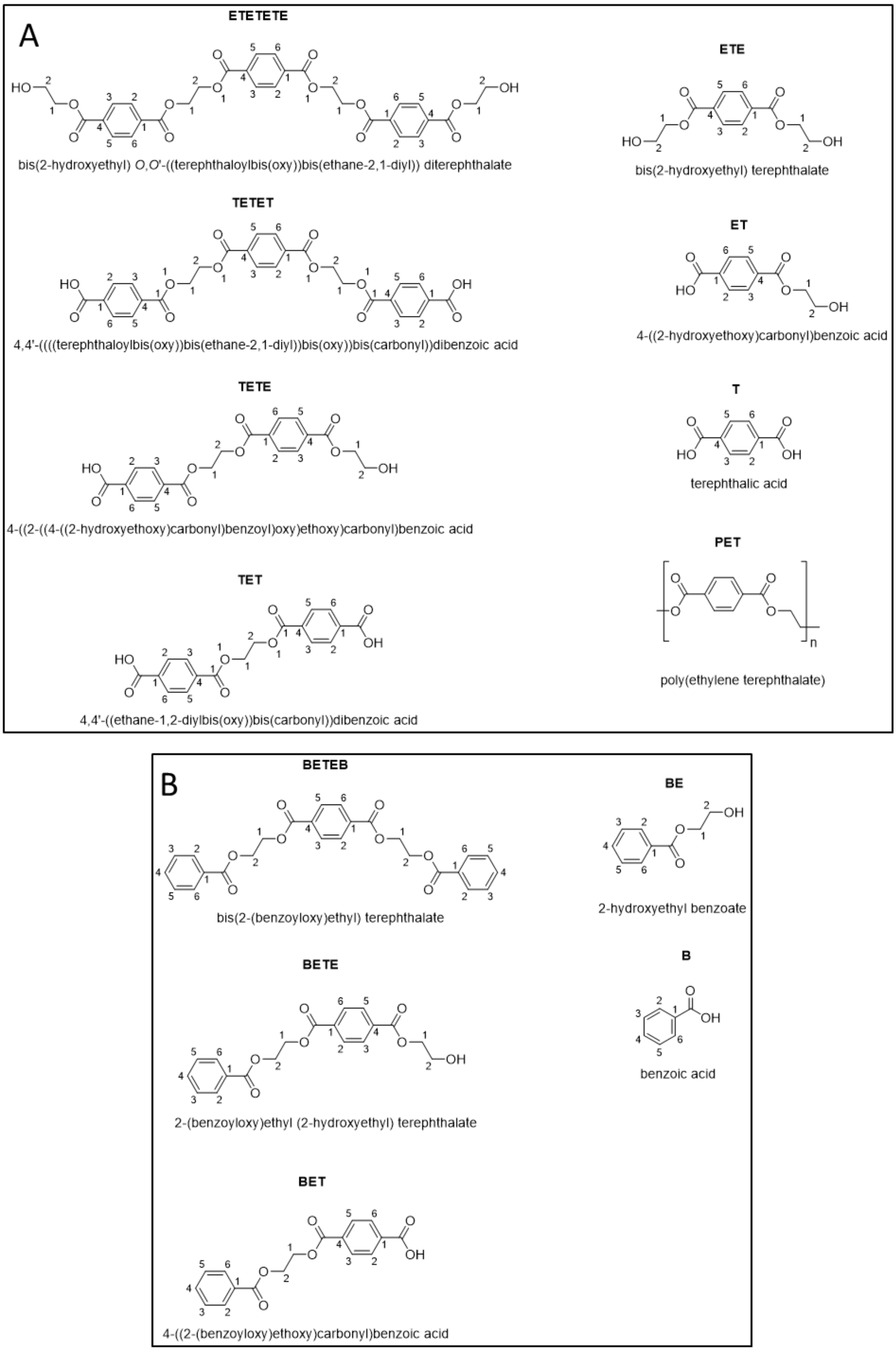

Figure S2. **Chemical structures, abbreviations and full chemical names of PET and PET model compounds** used as substrates in enzyme reactions and/or standard samples in RP-HPLC analyzes. Box A contains PET and possible degradation products, whereas box B contains the model substrate BETEB and degradation products. It should be noted that T, ET and ETE are possible reaction products from both PET and BETEB hydrolysis.

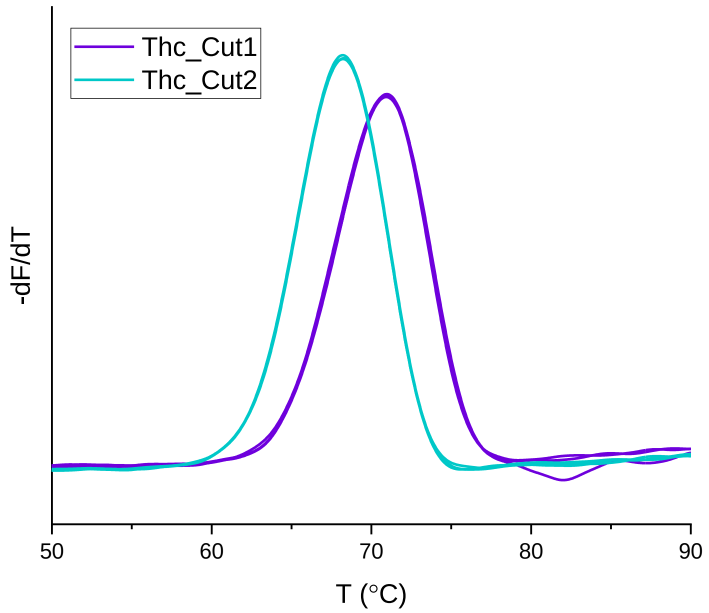

Figure S3. Melt curves derived from the 350/330 nm fluorescence ratio (overlay of triplicate measurements) showing T_m values of 71 and 68 °C for Thc_Cut1 (purple) and Thc_Cut2 (turquoise), respectively.

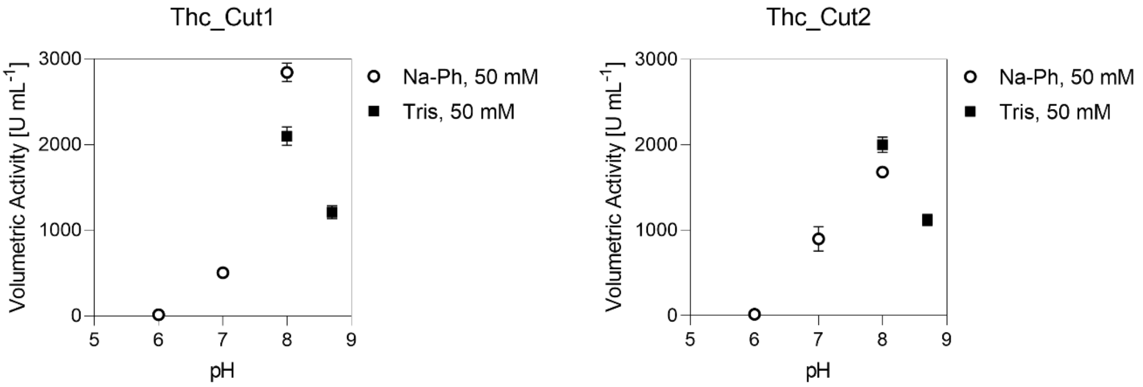

Figure S4. pH profile of Thc_Cut1 and Thc_Cut2. Activities were measured against pNP-C8 at 3.125 mM substrate concentration. Sodium phosphate (Na-Ph) and tris-Hcl (Tris) buffers were used at 50 mM concentration, as indicated. Data represent mean values from duplicate experiment, error bars indicate the spread.

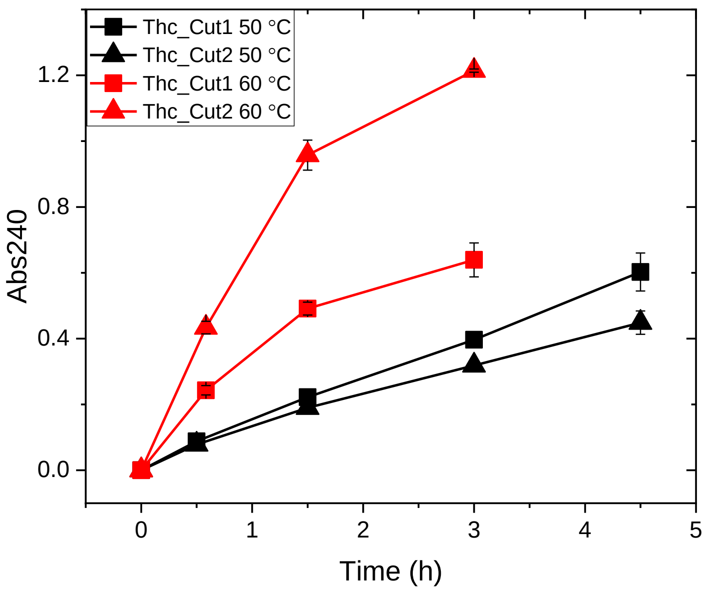

Figure S5. Progress curves from enzymatic PET hydrolysis for Thc_Cut1 (squares) and Thc_Cut2 (triangles) at 50 °C (red) or 60 °C (black) over 4.5 h. The graph represents curves when PET is in excess (20 g/L PET, 0.1 µM enzyme). Data point represent mean values of duplicate experiments, error bars indicate the spread. MM analyzes were conducted within the time of the linear range for respective enzyme and temperature (steady-state).

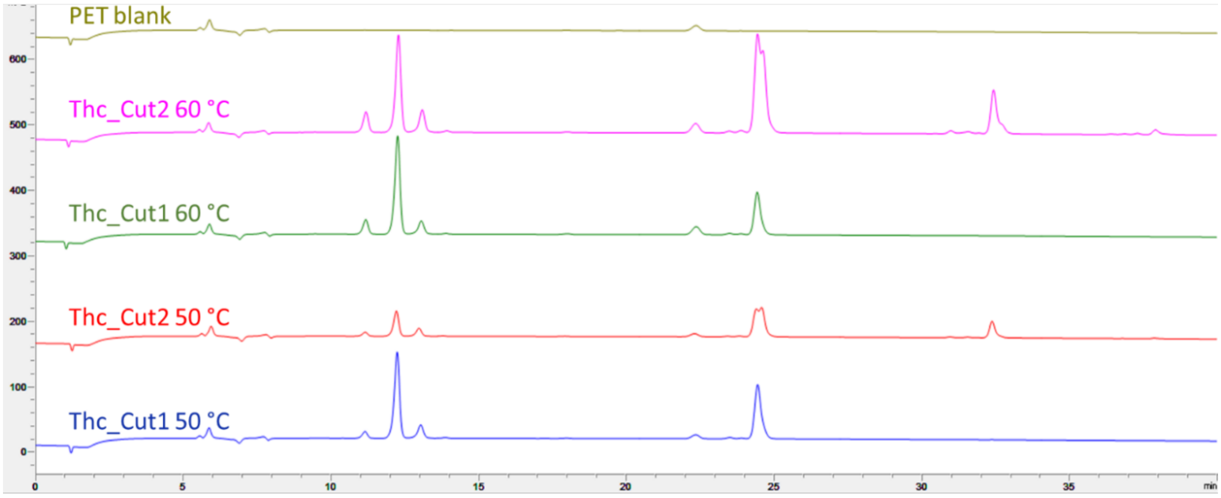

Figure S6. Product profiles from RP-HPLC analysis of enzymatic PET hydrolysis over 3 h at 50 or 60 °C with 20 g L^{-1} PET and 0.1 µM enzyme. Major peaks in the product profile correspond to (from left to right): T, ET, ETE (between 11-13 min), TET/E (species with two aromatic rings at 24 min) and E/TETET/E (species with three aromatic rings, only seen in Thc_Cut2 reactions at 33 min).

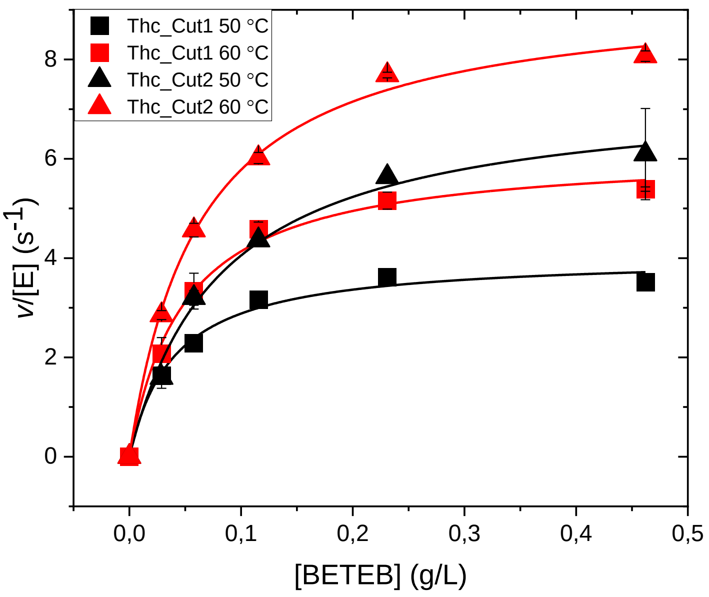

Figure S7. MM plots for Thc_Cut1 (squares) and Thc_Cut2 (triangles) with initial hydrolysis rate as a function of BETEB concentration. Symbols are experimental data from 15-20 min reactions at 50 °C (black) or 60 °C (red) with 0.005 and 0.01 µM enzyme for Thc_Cut2 and Thc_Cut1, respectively. Data points represent the mean value of duplicate measurements, the error bars indicate the spread. Lines represent the best fit of the non-linear MM equation.

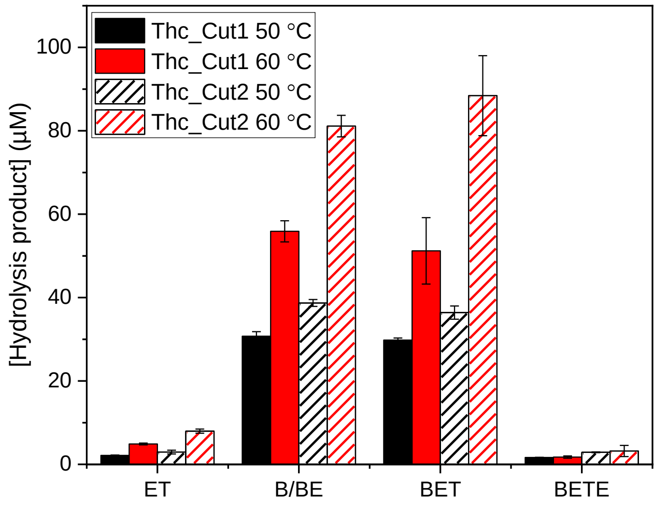

Figure S8. Product quantification from RP-HPLC analysis of BETEB hydrolysis by ThC_Cut1 (filled bars) and ThC_Cut2 (striped bars) over 20 min at 50 (black) or 60 °C (red) with 0.5 g L^{-1} BETEB and 0.01 µM enzyme. Data represent the mean value of duplicate measurements, the error bars indicate the spread.

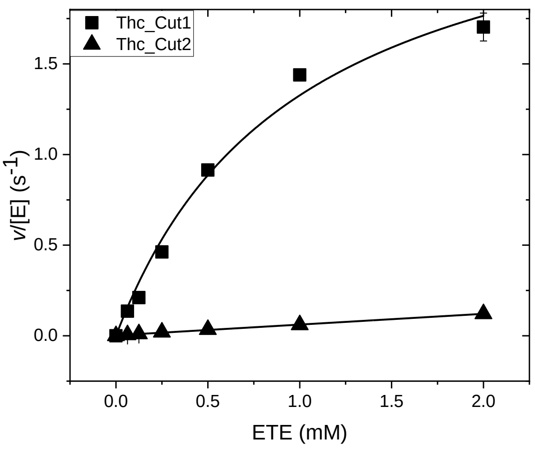

Figure S9. MM plots for Thc_Cut1 (squares) and Thc_Cut2 (triangles) with initial hydrolysis rate as a function of ETE concentration. Symbols are experimental data from 15 min reactions at 50 °C with 0.5 and 0.1 µM enzyme for Thc_Cut2 and Thc_Cut1, respectively. Data point represent the mean value of duplicate measurements, the error bars indicate the spread. Data for Thc_Cut1 are fitted with the non-linear MM equation whereas data for Thc_Cut2 could not be saturated up to 2 mM ETE and therefore fitted with linear regression.

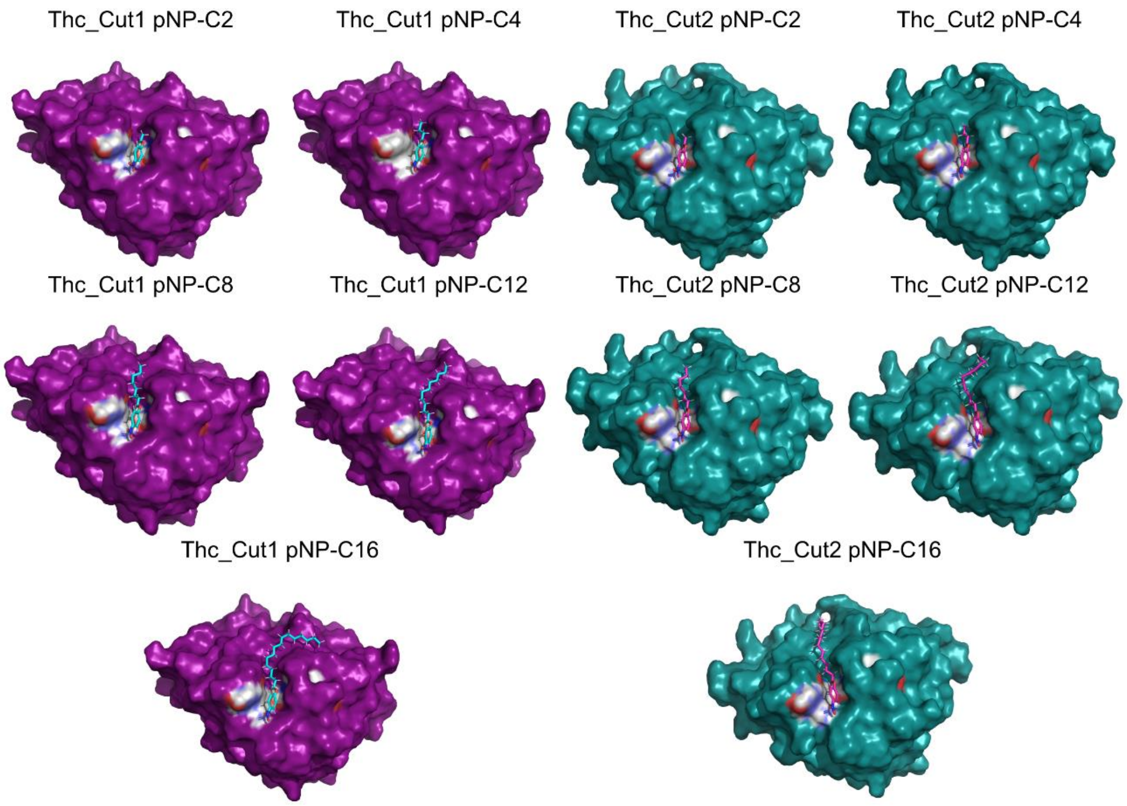

Figure S10. Thc_Cut1 and Thc_Cut2 docked with pNP-C2 to C16. Enzymes and ligands as indicated in the panels.

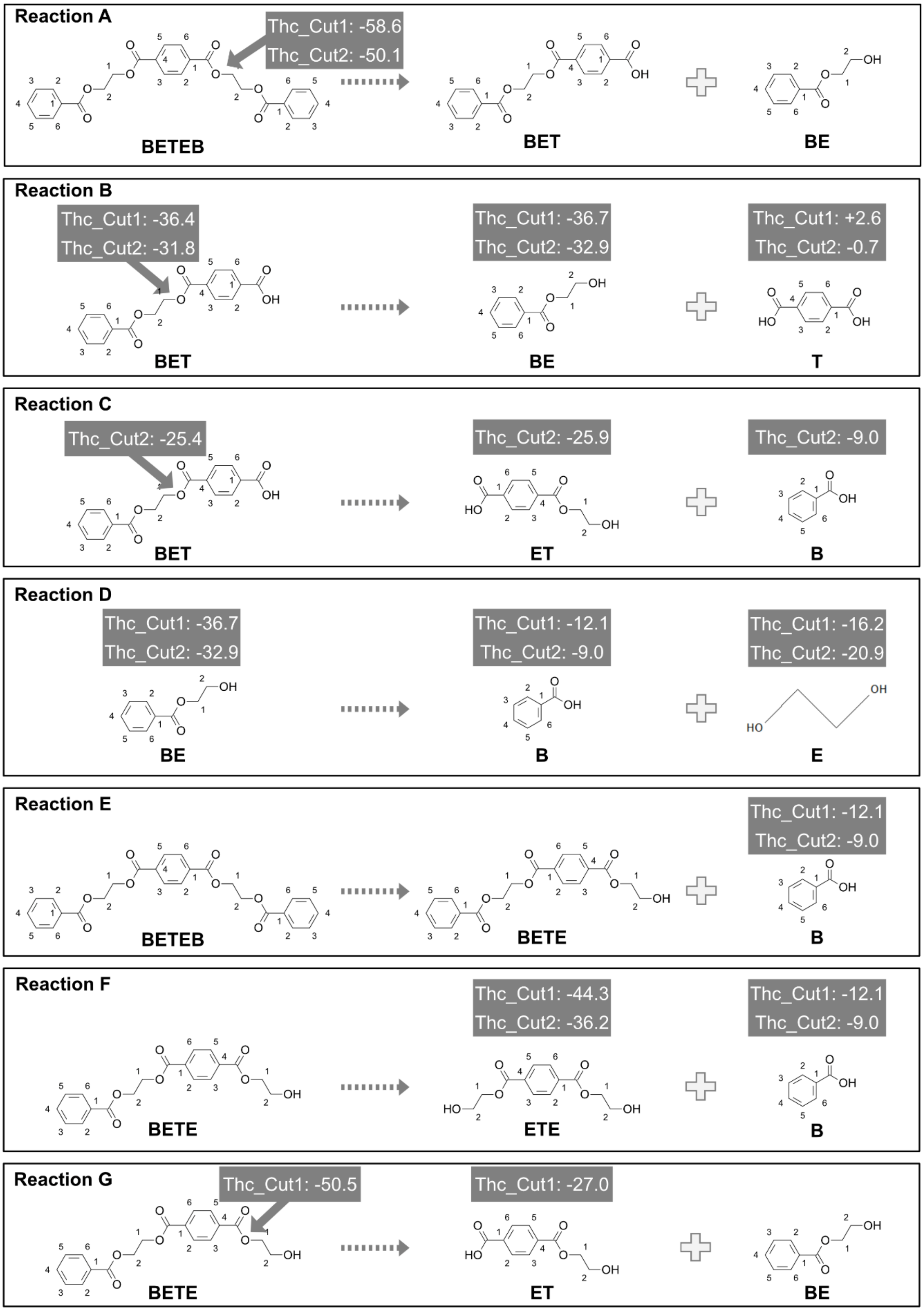

Figure S11. Reactions involved in BETEB degradation by Thc_Cut1 and Thc_Cut2. Results for docking analysis were added where possible and numbers are stating the binding energies in kcal mol^{-1}. Binding energies and the corresponding docking scores are given in Table S3.

Table S1. Overview of amino acid differences and their location/function in Thc_Cut1 and Thc_Cut2.

	Surface	"Region 1"	Thc_Cut2 longer residue
Amino acid differences	Ser/Arg19	Asn/Arg29	Ser/Arg19
Thc_Cut1/Thc_Cut2	Asn/Arg29	Val/Arg30	Asn/Arg29
	Leu/Phe33	Leu/Phe33	Leu/Phe33
	Ser/Gly34	Ser/Gly34	Ser/Asp36
	Ser/Asp36	Ser/Asp36	Ile/Asn88
	Glu/Gln65	Glu/Gln65	Glu/Arg99
	Ile/Asn88	Ile/Val68	Thr/Arg166
	Glu/Arg99	Ile/Asn88	Ala/Leu183
	Asn/Asp106	Glu/Arg99	
	Arg/Asp111	Asn/Asp106	
	Thr/Ala115	His/Tyr107	
	Thr/Arg166	Arg/Asp111	
	Ala/Leu183	Thr/Ala115	
	Lys/Arg187		
	Ser/Thr195		

Table S2. Results of docking analyzes of Thc_Cut1 and Thc_Cut2 with BETEB, ETE and pNP-C2 to C16[a].

	Binding Energy [kcal mol^{-1}]	Docking Score
	**Thc_Cut1**	
BETEB	-58.58	-5.50
ETE	-44.34	-2.93
pNP-C2	-36.51	-4.42
pNP-C4	-42.96	-4.24
pNP-C8	-51.85	-2.59
pNP-C12	-59.64	-4.74
pNP-C16	-67.03	-4.90
	**Thc_Cut2**	
BETEB	-50.12	-4.77
ETE	-36.82	-1.46
pNP-C2	-35.76	-4.55
pNP-C4	-42.75	-4.75
pNP-C8	-51.81	-2.70
pNP-C12	-55.23	-4.86
pNP-C16	-61.34	-4.12

a) Docking was performed with the Schrödinger Maestro software. All information on protein and ligand preparation, as well as chosen constraints can be found under Experimental procedures in the main article.

Table S3. Results of docking analyzes of Thc_Cut1 and Thc_Cut2 with BETEB and its degradation products[a)]

	Binding Energy [kcal mol^{-1}]	Docking Score
**Thc_Cut1**		
BETEB	-58.58	-5.50
BETE	-50.45	-6.20
BET	-36.37	-6.16
BE	-35.72	-4.80
ETE	-44.34	-2.93
T	2.59	-5.38
EG	-16.21	-2.24
B	-12.07	-4.89
ET	-26.96	-4.85
**Thc_Cut2**		
BETEB	-50.12	-4.77
BETE	*No pose*	
BET-1	-31.79	-4.73
BET-2	-25.43	-3.68
BE	-32.91	-4.59
ETE	-36.82	-1.46
T	-0.70	-5.27
EG	-20.94	-2.11
B	-9.03	-5.11
ET	-25.93	-4.58

a) Docking was performed with the Schrödinger Maestro software. Reactions are visualized in Figure S11. All information on protein and ligand preparation, as well as chosen constraints can be found in the materials and methods section in the main article.
